# Immune checkpoint inhibitor-related myositis with hepatitis in HER2-positive metastatic breast cancer after trastuzumab deruxtecan plus sintilimab: a case report

**DOI:** 10.3389/fonc.2026.1888036

**Published:** 2026-07-28

**Authors:** Ying Long, Xun Xiao, Xinyu Ji, Zheng Wang, Haiyan Mao, Shenxiang Liu

**Affiliations:** 1 Department of Oncology, The Affiliated Hospital of Yangzhou University, Yangzhou University, Yangzhou, Jiangsu, China; 2 Department of Pathology, The Affiliated Hospital of Yangzhou University, Yangzhou University, Yangzhou, Jiangsu, China

**Keywords:** antibody-drug conjugate, hepatitis, HER2-positive breast cancer, immune checkpoint inhibitor, immune-related myositis, ocular involvement, sintilimab, trastuzumab deruxtecan

## Abstract

Immune checkpoint inhibitors (ICIs) have improved outcomes across multiple solid tumors, but they may trigger immune-related adverse events involving the nervous system, skeletal muscle, myocardium, and liver. ICI-related myositis is uncommon but clinically important and may overlap with myocarditis or other neuromuscular toxicities. Reports in breast cancer remain limited, particularly in HER2-positive disease outside standard immunotherapy settings. We describe a 67-year-old woman with HER2-positive metastatic breast cancer who had received multiple anti-HER2 regimens and had limited tolerance to chemotherapy and endocrine therapy. After multidisciplinary discussion and informed consent, her regimen was adjusted to trastuzumab deruxtecan plus sintilimab because of progressive pulmonary and intracranial disease. After the first cycle, she developed acute bilateral ptosis with mild bilateral lower-extremity weakness, marked creatine kinase elevation (peak CK 8075.3 U/L), CK-MB elevation (206.4 U/L), and severe transaminase elevation. Repetitive nerve stimulation did not show a typical decremental response, left orbicularis oculi fatigue testing was negative, myasthenia gravis-related antibody testing was recommended but declined, and the response to cholinesterase inhibitor therapy was limited. Troponin testing, repeat echocardiography, and cardiac magnetic resonance imaging were not performed. Therefore, the condition was reinterpreted as immune-related myositis with predominant ocular involvement and concomitant hepatitis; concomitant myasthenia gravis remained unproven and myocarditis could not be definitively excluded. Sintilimab was discontinued, and high-dose methylprednisolone was initiated and tapered. Muscle and liver enzymes decreased substantially, and ptosis partially improved. The patient received no further anticancer treatment and died in December 2025; telephone follow-up suggested progressive metastatic breast cancer as the most likely cause. This case highlights that new ocular symptoms accompanied by marked CK elevation after PD-1 inhibitor-containing therapy should prompt early consideration of immune-related myositis, even when classical myasthenia gravis is not supported by the available evidence.

## Introduction

Breast cancer is the most frequently diagnosed malignancy in women worldwide (1). Immune checkpoint inhibitors targeting the PD-1/PD-L1 axis have become part of standard treatment in several malignancies and selected triple-negative breast cancer settings (2–5). In contrast, systemic treatment for HER2-positive metastatic breast cancer still centers on sequential anti-HER2 therapy. Although immunotherapy is not a routine guideline-defined component of HER2-positive metastatic breast cancer treatment, individualized ICI-containing strategies may occasionally be considered after multidisciplinary evaluation when standard options are exhausted and disease continues to progress.

Neurologic and muscular immune-related adverse events are uncommon but clinically important because of their potentially rapid progression and life-threatening course. ICI-related myositis may present with ocular, bulbar, respiratory, limb, or cardiac involvement and may be difficult to distinguish from myasthenia gravis when ocular symptoms predominate (6–12). We report a case of immune-related myositis with predominant ocular involvement and concomitant hepatitis after trastuzumab deruxtecan plus sintilimab in a woman with HER2-positive metastatic breast cancer, emphasizing diagnostic uncertainty, oncologic context, and the clinical implications of antibody-drug conjugate (ADC) plus PD-1 blockade.

## Case presentation

### Patient history and oncologic background

A 67-year-old woman underwent modified radical mastectomy for right breast cancer in December 2018. Postoperative pathology showed moderately differentiated invasive carcinoma measuring approximately 2.0 x 2.0 x 2.0 cm, with no metastasis in 18 axillary lymph nodes. Immunohistochemistry showed ER 20% positivity, PR negativity, HER2 1+, CK7 positivity, CK20 negativity, Villin negativity, SMMHC negativity, calponin negativity, E-cadherin positivity, and Ki-67 approximately 3%. The pathologic stage was IIA (pT2N0M0).

Although HER2 immunohistochemistry in the primary breast tumor was scored as 1+, HER2 gene amplification was confirmed by fluorescence *in situ* hybridization (FISH), establishing HER2-positive disease.

**Figure 1:**
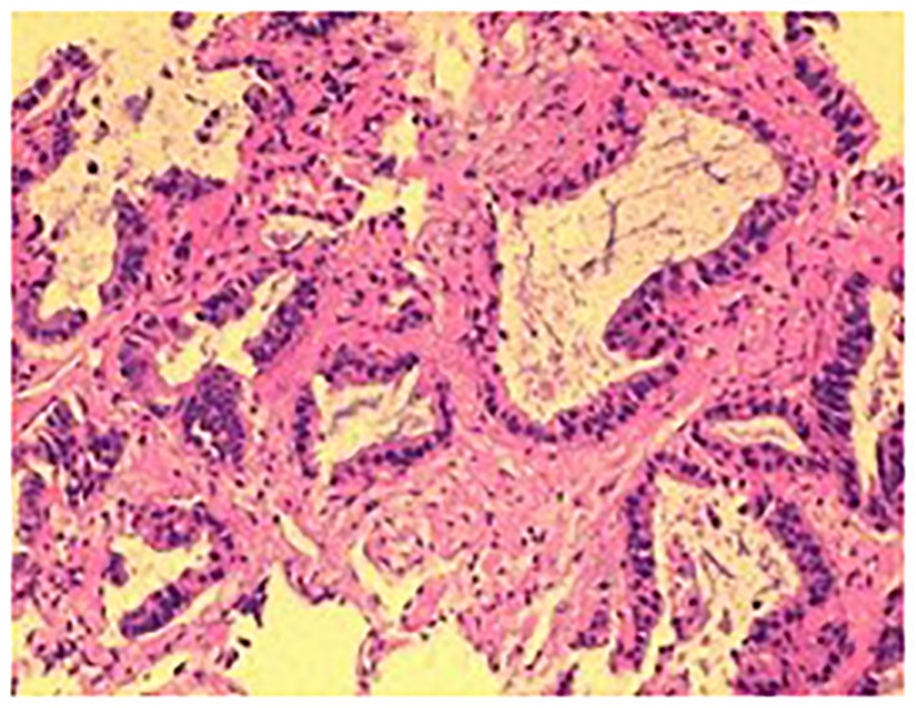
Hematoxylin–eosin staining of the pulmonary metastatic lesion confirming metastatic breast invasive ductal carcinoma.

**Figure 2:**
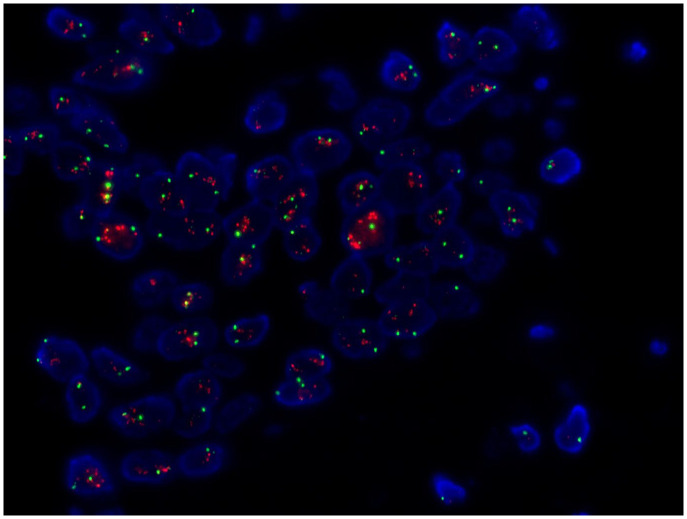
HER2 gene amplification demonstrated by fluorescence *in situ* hybridization (FISH) in the pulmonary metastatic lesion.

She received six cycles of adjuvant docetaxel plus pirarubicin from December 2018 to April 2019. Adjuvant endocrine therapy with anastrozole and later letrozole was discontinued because of intolerance, including headache and generalized pain. In December 2022, chest and abdominal computed tomography demonstrated multiple pulmonary nodules. CT-guided lung biopsy confirmed metastatic invasive ductal carcinoma of breast origin (**Figure 1**). Immunohistochemistry showed ER approximately 90% strong positivity, PR negativity, and HER2 2+, with HER2 amplification confirmed by FISH (**Figure 2**).

Thus, HER2 amplification was maintained in the pulmonary metastatic lesion, although HER2 immunohistochemical expression changed from 1+ in the primary tumor to 2+ in the metastatic lesion.

The patient subsequently received multiple systemic therapies. A detailed summary of the oncologic treatment history and reasons for treatment modification is provided in **Supplementary Table 1**. In January to March 2023, she received trastuzumab combined with albumin-bound paclitaxel and cisplatin, but chemotherapy was complicated by chest tightness, dyspnea, fatigue, anorexia, and grade III myelosuppression, leading to treatment interruption. She then received trastuzumab-based maintenance therapy. Further systemic treatment included capecitabine plus pyrotinib, tamoxifen, trastuzumab emtansine, and trastuzumab deruxtecan; treatment changes were driven by disease progression or intolerance.

Bone and brain metastases were documented in 2024. She received whole-brain radiotherapy in May 2024 and bone-modifying therapy. Trastuzumab emtansine was administered from August to November 2024. In February 2025, imaging showed progression of pulmonary and intracranial metastases, and trastuzumab deruxtecan was initiated on 7 February 2025 and continued on 3 March, 31 March, and 28 April 2025.

**Figure 3:**
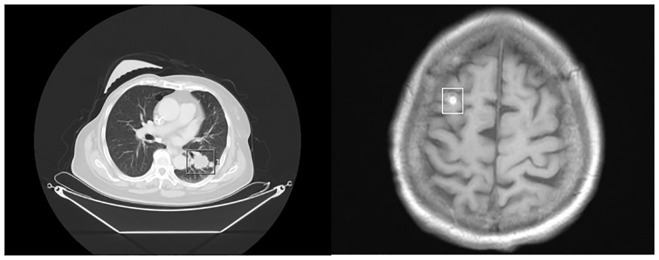
Radiological progression of metastatic disease before initiation of trastuzumab deruxtecan plus sintilimab.

On 5 June 2025, CT-guided biopsy of a left lower lung lesion again confirmed metastatic breast invasive ductal carcinoma. Immunohistochemistry showed CKpan positivity, CK20 negativity, TTF-1 negativity, Napsin A negativity, GATA3 positivity, CDX-2 negativity, Villin positivity, Ki-67 approximately 40%, ER approximately 5%, PR approximately 5%, HER2 2+, and TRPS1 positivity. Brain MRI showed post-radiotherapy changes with multiple intracranial metastases, some newly detected and some slightly enlarged compared with the previous examination (**Figure 3**).

The re-biopsy also demonstrated substantial changes in hormone receptor expression, highlighting receptor conversion during heavily pretreated metastatic disease.

Because the patient had progressive HER2-positive metastatic breast cancer after multiple anti-HER2 regimens and had limited tolerance to chemotherapy and endocrine therapy, the multidisciplinary team recommended an individualized later-line regimen of trastuzumab deruxtecan plus sintilimab. PD-L1 expression, tumor mutational burden, and MSI/MMR status were not assessed before sintilimab initiation. The potential benefits and immune-related risks, including myocarditis, thyroiditis, hepatitis, nephritis, enteritis, hypophysitis, skin reactions, and potentially fatal immune toxicity, were explained to the patient and her family. Written informed consent was obtained for the individualized regimen and for publication of this case report with de-identified clinical information.

On 11 June 2025, treatment was changed to trastuzumab deruxtecan 200 mg every 3 weeks plus sintilimab 200 mg every 3 weeks, with ongoing bone-modifying therapy.

### Baseline evaluation before the ICI-containing regimen

During the hospitalization immediately preceding the ICI-containing regimen, laboratory tests on 3 June 2025 showed leukopenia (white blood cell count 3.23 x 10^9/L), hemoglobin 119 g/L, and platelet count 114 x 10^9/L. Liver function tests were not severely abnormal at that time, with ALT 13.9 U/L and AST 26.8 U/L. Electrocardiography was reported as normal. There was no clinical or laboratory evidence of active muscle injury before the sintilimab-containing regimen. Tumor markers were elevated, including CEA 307.98 ng/mL and CA15-3 215.00 U/mL.

### Diagnostic assessment and laboratory details during toxicity

Approximately four days before admission on 14 July 2025, the patient developed acute bilateral ptosis. She had no clear fluctuating symptoms or diurnal variation. She denied diplopia, dysphagia, dysarthria, and dyspnea, but reported mild bilateral lower-extremity weakness. Ice-pack testing was not performed.

Laboratory tests on 14 July 2025 revealed marked abnormalities: ALT 449.0 U/L, AST 547.5 U/L, gamma-glutamyltransferase 70.8 U/L, alkaline phosphatase 177.1 U/L, lactate dehydrogenase 1439.4 U/L, CK 8075.3 U/L, CK-MB 206.4 U/L, alpha-hydroxybutyrate dehydrogenase 1106.9 U/L, and high-sensitivity C-reactive protein 15.03 mg/L. These findings provided strong biochemical evidence of immune-related myositis and concomitant hepatitis. Electrocardiography showed sinus tachycardia, left anterior fascicular block, clockwise rotation, and ST-segment changes in V1-V3. Troponin testing, repeat echocardiography, and cardiac magnetic resonance imaging were not performed; therefore, myocarditis could not be definitively confirmed or excluded.

Neurologic consultation was obtained. Nerve conduction studies showed that the tested motor and sensory nerve conduction velocities and amplitudes were within the normal or borderline range, and F-wave latency was normal. Low- and high-frequency repetitive nerve stimulation did not show a significant decrement or increment in compound muscle action potential amplitude, and left orbicularis oculi fatigue testing was negative. Testing for myasthenia gravis-related antibodies, including anti-acetylcholine receptor and anti-MuSK antibodies, was recommended, but the patient and her family declined further serologic evaluation. Single-fiber electromyography was not performed.

Although myasthenia gravis was considered in the differential diagnosis because of acute bilateral ptosis, the absence of typical fluctuating symptoms, negative electrophysiological findings, lack of serological confirmation, and limited response to acetylcholinesterase inhibitor therapy did not support a diagnosis of myasthenia gravis. Given the temporal relationship with sintilimab exposure, acute bilateral ptosis, mild lower-extremity weakness, marked muscle enzyme elevation, severe transaminase elevation, and improvement after immunotherapy withdrawal and corticosteroid therapy, the clinical picture was reinterpreted as immune-related myositis with predominant ocular involvement and concomitant hepatitis.

### Therapeutic intervention and follow-up

Sintilimab was discontinued immediately after the immune-related neuromuscular syndrome was recognized. Intravenous methylprednisolone was initiated at 160 mg/day on 14 July 2025 and gradually tapered to 120 mg/day, 80 mg/day, and 60 mg/day according to clinical and laboratory response. Cholinesterase inhibitor therapy was also used for ptosis, but the clinical response was limited.

After corticosteroid therapy, liver enzymes and muscle enzymes decreased substantially. The dynamic changes in major laboratory parameters are summarized in **Supplementary Table 2**. CK decreased from 8075.3 U/L to 971.9 U/L on 17 July and to 281.5 U/L on 24 July. ALT decreased from 449.0 U/L to 271.9 U/L on 17 July and to 81.8 U/L on 24 July. AST decreased from 547.5 U/L to 108.9 U/L on 17 July and to 46.5 U/L on 24 July. Bilateral ptosis improved but did not completely resolve. The patient was discharged with oral methylprednisolone tapering and outpatient follow-up.

**Figure 4:**
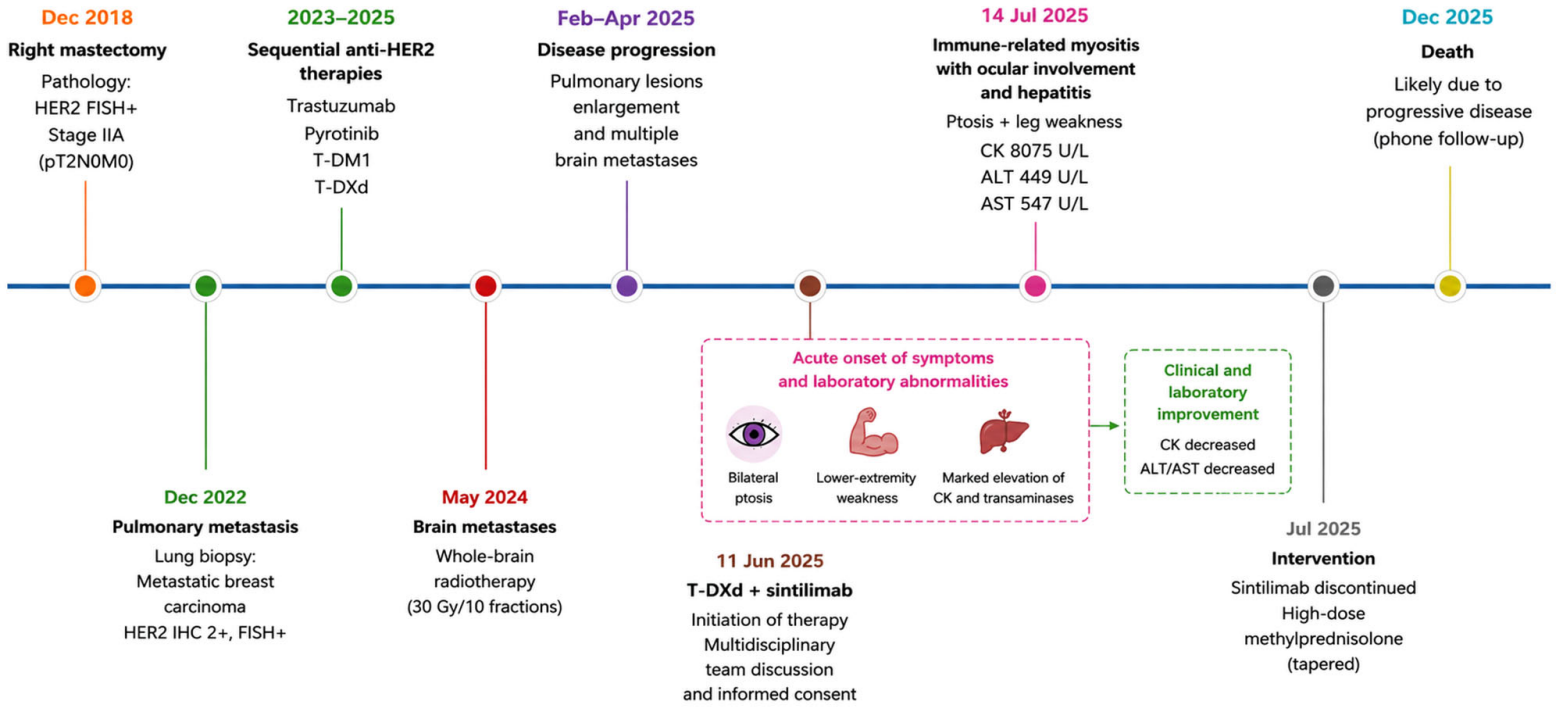
Clinical timeline of oncologic treatment, immune-related myositis with ocular involvement and hepatitis, and therapeutic response.

At neurologic outpatient follow-up on 19 August 2025, persistent ptosis was noted, and the response to cholinesterase inhibitor therapy was limited. The patient received no further anticancer treatment after this adverse event. She died in December 2025 according to telephone follow-up information obtained from family members. Although the exact cause of death could not be verified, progressive metastatic breast cancer was considered the most likely cause. There was no evidence suggesting ongoing neuromuscular toxicity as the direct cause of death. The overall clinical course, oncologic treatment sequence, development of immune-related adverse events, and response to corticosteroid therapy are summarized in **Figure 4**.

## Discussion

This case describes immune-related myositis with predominant ocular involvement and concomitant hepatitis after trastuzumab deruxtecan plus sintilimab in a patient with heavily pretreated HER2-positive metastatic breast cancer. The most important feature of this case is not simply the occurrence of neuromuscular immune-related toxicity, but its development in a non-standard, individualized later-line treatment setting involving an ADC combined with a PD-1 inhibitor.

The diagnosis required cautious interpretation. Although acute bilateral ptosis initially raised concern for myasthenia gravis, the absence of typical fluctuating symptoms, lack of diurnal variation, negative repetitive nerve stimulation, negative orbicularis oculi fatigue testing, absence of serological confirmation, and limited response to acetylcholinesterase inhibitor therapy collectively weaken support for a diagnosis of myasthenia gravis. In contrast, the marked elevation of CK, CK-MB, LDH, and transaminases, together with lower-extremity weakness and substantial improvement after corticosteroid therapy, strongly support immune-related myositis with concomitant hepatitis. Therefore, immune-related myositis with predominant ocular involvement represents the most appropriate interpretation of this case.

The differential diagnosis included immune-related myositis with ocular involvement, classical ocular myasthenia gravis, brain metastasis-related ocular symptoms, paraneoplastic neuromuscular disease, and drug-related muscle injury. Brain metastases were present, but the abrupt onset of bilateral ptosis after immunotherapy, marked CK elevation, lower-extremity weakness, and improvement after corticosteroid therapy favored immune-mediated myositis. Accordingly, myasthenia gravis is discussed as a differential diagnosis rather than as the final diagnosis.

ICI-related myositis, myocarditis, and myasthenia gravis may occur within an overlapping neuromuscular toxicity spectrum (6–12). In this patient, CK-MB and LDH were markedly elevated, and electrocardiography showed sinus tachycardia with conduction and ST-segment changes. However, troponin testing, repeat echocardiography, and cardiac magnetic resonance imaging were not performed. Thus, myocarditis could not be definitively confirmed or excluded, and the case should not be presented as definite myocarditis-overlap syndrome.

From an oncologic perspective, this case is clinically relevant because immunotherapy is not a standard routine approach in HER2-positive metastatic breast cancer. The most robust evidence for ICIs in breast cancer remains concentrated in triple-negative breast cancer (4, 13). HER2-positive metastatic breast cancer is primarily managed with sequential anti-HER2 therapy. Trastuzumab deruxtecan has shown substantial efficacy in HER2-positive metastatic breast cancer in trials such as DESTINY-Breast03 and DESTINY-Breast02 (5, 14). In the present case, however, the patient had already received multiple anti-HER2 regimens and continued to have progressive pulmonary and intracranial disease. The decision to add sintilimab was individualized rather than guideline-standard. PD-L1 expression, TMB, and MSI/MMR status were not assessed, limiting interpretation of the biological rationale for immunotherapy.

Causality should also be discussed in a balanced manner. Sintilimab was the most likely trigger of the immune-related adverse event because severe muscle and liver enzyme abnormalities developed after initiation of the sintilimab-containing regimen and improved after its discontinuation and corticosteroid therapy. However, trastuzumab deruxtecan was administered concurrently, and the adverse event should not be attributed exclusively to sintilimab with absolute certainty. ADC plus ICI combinations may have toxicity profiles that differ from either agent alone. T-DXd is also associated with important adverse events, particularly interstitial lung disease/pneumonitis, which remains a clinically significant and potentially fatal toxicity (15).

As ADC-immunotherapy combinations are increasingly being investigated across breast cancer subtypes, including sacituzumab govitecan plus pembrolizumab in advanced triple-negative breast cancer (16), clinicians should remain alert not only to common treatment-related adverse events but also to rare severe neuromuscular immune-related toxicities. This case suggests that new ptosis accompanied by marked CK elevation after PD-1 inhibitor-containing therapy should prompt immediate evaluation for immune-related myositis with possible ocular involvement, rather than being attributed automatically to myasthenia gravis.

This case also illustrates how severe immune-related toxicity may affect the overall oncologic trajectory. Although ptosis and laboratory abnormalities improved after corticosteroid treatment, the patient did not receive further anticancer therapy and died several months later. The available data do not support direct attribution of death to neuromuscular toxicity. Nevertheless, the adverse event interrupted systemic therapy in a patient with advanced metastatic disease and may have indirectly contributed to subsequent oncologic decline.

## Limitations

Several limitations should be acknowledged. First, myasthenia gravis-related antibody testing and single-fiber electromyography were not performed because the patient declined further evaluation. Therefore, concomitant myasthenia gravis could not be definitively assessed. However, the currently available evidence is insufficient to support a diagnosis of myasthenia gravis.

Second, troponin measurements, repeat echocardiography, and cardiac magnetic resonance imaging were unavailable; therefore, myocarditis could not be definitively excluded. Third, PD-L1 expression, tumor mutational burden, and MSI/MMR status were not assessed before initiation of sintilimab. Finally, the exact cause of death could not be confirmed because follow-up information was obtained by telephone, although progressive metastatic breast cancer was considered the most likely explanation.

Accordingly, the final diagnosis is best interpreted as immune-related myositis with predominant ocular involvement and concomitant hepatitis, while concomitant myasthenia gravis remains unproven.

## Conclusion

This case is best interpreted as immune checkpoint inhibitor-related myositis with predominant ocular involvement and concomitant hepatitis occurring after trastuzumab deruxtecan plus sintilimab in a heavily pretreated patient with HER2-positive metastatic breast cancer. The marked elevation of muscle enzymes, severe transaminase abnormalities, lower-extremity weakness, and favorable response to corticosteroid therapy strongly supported immune-related myositis and hepatitis. Clinicians should maintain a high index of suspicion for immune-related neuromuscular toxicity when new ocular symptoms occur after PD-1 inhibitor-containing therapy, even in the absence of definitive evidence for myasthenia gravis.

## Patient perspective

The patient and her family were informed that standard treatment options were limited because of repeated disease progression and intolerance to several previous therapies. After discussion with the multidisciplinary team, they agreed to the individualized regimen of trastuzumab deruxtecan plus sintilimab. When immune-related neuromuscular toxicity was suspected, the patient and her family accepted discontinuation of sintilimab and corticosteroid treatment, but declined further myasthenia gravis-related antibody testing.

## Data Availability

The data that support the findings of this study are available from the corresponding author, SL upon reasonable request.
