# Supplementary material for: Immune checkpoint inhibitor-related myositis with hepatitis in HER2-positive metastatic breast cancer after trastuzumab deruxtecan plus sintilimab: a case report
Source: Front Oncol. 2026 Jul 28;16:1888036. doi: 10.3389/fonc.2026.1888036 (PMC13457053; doi:10.3389/fonc.2026.1888036)
Supplement: Supplementary file 1 [file Table1.docx]

**Supplementary Table S1. Summary of major oncologic treatments and reasons for discontinuation.**

| **Period** | **Treatment** | **Clinical context / response** | **Reason for discontinuation or change** |
| --- | --- | --- | --- |
| Dec 2018-Apr 2019 | Docetaxel + pirarubicin | Adjuvant chemotherapy after surgery | Completed planned treatment |
| 2019 | Anastrozole, then letrozole | Adjuvant endocrine therapy | Intolerance |
| Jan-Mar 2023 | Trastuzumab + albumin-bound paclitaxel + cisplatin | Pulmonary metastatic recurrence | Chemotherapy intolerance |
| Mar 2023-Mar 2024 | Trastuzumab maintenance with additional systemic/endocrine strategies | Maintenance after initial metastatic therapy | Disease progression |
| Jun-Aug 2024 | Capecitabine + pyrotinib | Metastatic disease with brain/bone involvement | Disease progression |
| Jun 2024 | Tamoxifen | Endocrine therapy attempt | Intolerance |
| Aug-Nov 2024 | Trastuzumab emtansine (T-DM1) | Later-line anti-HER2 therapy | Disease progression |
| Feb-Apr 2025 | Trastuzumab deruxtecan (T-DXd) | Progressive HER2-positive metastatic disease | Disease progression |
| Jun 2025 | T-DXd + sintilimab | Individualized later-line regimen after MDT discussion | Immune-related adverse event |

Note: This supplementary table is provided to address the reviewers' request for a more detailed oncologic treatment history while keeping the main manuscript within the Frontiers Case Report display-item limit.
