# Supplementary material for: Immune checkpoint inhibitor-related myositis with hepatitis in HER2-positive metastatic breast cancer after trastuzumab deruxtecan plus sintilimab: a case report
Source: Front Oncol. 2026 Jul 28;16:1888036. doi: 10.3389/fonc.2026.1888036 (PMC13457053; doi:10.3389/fonc.2026.1888036)
Supplement: Supplementary file 2 [file Table2.docx]

**Supplementary Table S2. Dynamic laboratory changes during the immune-related adverse event and corticosteroid treatment.**

| **Parameter** | **Baseline (3 Jun 2025)** | **Peak toxicity (14 Jul 2025)** | **After corticosteroids (24 Jul 2025)** |
| --- | --- | --- | --- |
| CK (U/L) | 44.4 | 8075.3 | 281.5 |
| CK-MB (U/L) | 10.7 | 206.4 | — |
| ALT (U/L) | 13.9 | 449.0 | 81.8 |
| AST (U/L) | 26.8 | 547.5 | 46.5 |
| LDH (U/L) | Normal | 1439.4 | ↓ |
| hsCRP (mg/L) | 2.14 | 15.03 | ↓ |
